# Re-scoping ultradian rhythms in the context of metabolism

**DOI:** 10.3389/fphys.2024.1504879

**Published:** 2024-12-12

**Authors:** Daan R. van der Veen, Menno P. Gerkema

**Affiliations:** ^1^ Chronobiology Section, Faculty of Health and Medical Sciences, University of Surrey, Guildford, United Kingdom; ^2^ Energy and Sustainability Research Institute Groningen, Groningen Institute for Evolutionary Life Sciences, University of Groningen, Groningen, Netherlands; ^3^ Groningen Institute for Evolutionary Life Sciences, at the Faculty of Science and Engineering, University of Groningen, Groningen, Netherlands

**Keywords:** circadian, ultradian, metabolism, episodic, infradian, circhoral, pulsatile

## Abstract

Rapid, ultradian biological rhythms are only partly comparable to circadian (24-h) rhythms. Often, the ensuing expectations from this comparison are that 1) ultradian rhythms should be driven by discrete oscillators, 2) they are biochemically buffered, and 3) they must be functionally linked to extrinsic events and cycles. These three expectations are not always met, but perhaps there is an adaptive benefit to ultradian rhythms not meeting these expectations, which sets them functionally apart from circadian rhythms. In view of the extensive descriptions of the manifold types of ultradian rhythms across all biological levels (e.g., see papers in this research topic), it seems important to ask whether we should actively create a new set of definitions and expectations. To make a start, we here lay out some important questions we need to ask about ultradian rhythms. We then illustrate how these questions highlight one key area of exploration: the linked expression of ultradian rhythms in metabolism and behaviour and the role this plays in addressing a negative energy balance.

## Introduction

Broadly speaking, biological rhythms can and do play a critical part in life. Two basic principles seem to emerge: rhythms are recurrent temporal patterns that result in predictable parameters like amplitude, phase, and period, and these rhythms allow related processes to align or couple, whilst simultaneously segregating incompatible processes. The function of circadian (24-h) and other “circa” rhythms, such as circatidal, seasonal, and lunar rhythms, lies in coupling behaviour and physiology with external environmental rhythms such as the day–night, tidal, lunar, or seasonal cycles (Aschoff, 1981; Aschoff, 1984). For non-circa, ultradian rhythms with periods less than 24 h, which typically lack an external physical counterpart, the function seems to lie in coupling internal cycles or interactions between conspecifics. For instance, ultradian rhythms within the TCA/Krebs cycle optimise energy metabolism, whilst simultaneously ensuring low ROS production during DNA replication to maintain genomic integrity (Ahn et al., 2017). Importantly, these metabolic rhythms are not driven by the cell cycle and persist even when the circadian clock is absent (Yang et al., 2022), suggesting that this is a functional temporal relationship between independent rhythms rather than a simple hourglass mechanism driven by metabolic events.

The duration of the day–night cycle is nearly constant, and the circadian rhythms associated with it are therefore expressed with a fairly constant period. However, when the day–night cycle becomes difficult to perceive, as during the arctic summer and winter, reindeer exhibit “circadian arrhythmicity” (van Oort et al., 2005). Moreover, common voles (*Microtus arvalis*) can easily be switched from circadian to ultradian behavioural rhythms just by removing the running wheel (Gerkema et al., 1990), which subsequently suppresses the circadian expression of peripheral circadian clock genes (van der Veen et al., 2006). Importantly, these behavioural foraging rhythms are driven by clocks, not homeostatic food or sleep drivers (Gerkema and van der Leest, 1991). The unmasking of ultradian rhythms is even clearer in non-natural conditions such as after genetic perturbations of circadian clocks (Vitaterna et al., 1994; Bunger et al., 2000; Zheng et al., 2001; Aviram et al., 2021; Ballance and Zhu, 2021).

These examples of modulations of the prominence of ultradian behavioural rhythms in direct response to changes in the external or internal 24-h rhythms pose the question whether this is a functional aspect of ultradian rhythms rather than an erratic characteristic of a weak clock. The requirement for circadian rhythms to be expressed continuously with a 24-h period is in our view a consequence of their functional coupling to the day–night cycle. In absence of such an abiotic counterpart for most ultradian rhythms, perhaps a better expectation is that ultradian rhythms are biological rhythms expressed when their coupled internal physiology is driving them to do so.

In an earlier example of cellular metabolism, how would ultradian rhythms in the TCA/Krebs cycle be affected if the need for glucose oxidation increased or decreased or if metabolic processing alters for any other reason? There are examples of ultradian behavioural rhythms being enhanced when voles experience a negative energy balance (van Rosmalen and Hut, 2021) and even in gene expression under hypoglycaemic conditions in cell culture (Ting et al., 2023). Not only the prominence of expression but also the period can be affected when, for instance, the midbrain dopamine tone is experimentally altered (Blum et al., 2014). These examples of “episodic” and/or “non-stationary” expressions of ultradian rhythms show that these rhythms are much more plastic than circadian rhythms. The observation that these may be evoked by physiological changes further suggests that there may be an adaptive benefit in this flexibility to respond to changes, e.g., seasonal or diet-induced changes in energy metabolism; however, very few studies exist that test this hypothesis. Experimentally, closing this gap (rather than inferring it from circadian rhythms) is critical to our definitions of the functions, relevance, and mechanisms of ultradian timing in behaviour and physiology.

## How do we classify the plethora of ultradian rhythms (the period questions)?

The original definition of ultradian rhythms—those rhythms with a period shorter than the circadian range (Halberg, 1965)—leaves us with a wide scatter of such rhythmic phenomena in frequency and biological background. Aschoff and Gerkema (1985) that the one unifying principle of ultradian rhythms is the variability in mechanisms and functions, valid as it still may be, is also what makes this group of biological rhythms so elusive. Yet, there may be classes of ultradian rhythms that sit in the same biological compartments and are expressed with similar periods. Some of these arbitrary groups, several of which are covered in this research topic, include the following:

• Neurobiological processes in the range of milliseconds to seconds (brain electrical dynamics (EEG), sinus node/heartbeat).
• Cellular biochemical homeostasis processes in the minutes to hours range (gene transcription activation, protein half-life, ATP, and cellular respiration (Murray et al., 2001; Brodsky, 2014)).
• Hormone-related rhythms such as HPA-axis activity and thermoregulation (Clarkson et al., 2017; Lightman et al., 2020; Grant and Kriegsfeld, 2023).
• Sleep processes in the 90-min range in humans [but this period differs between species] (NREM-REM, BRAC, and hemispheric dominance (Kleitman, 1982; Shannahoff-Khalsa, 1993)).
• Behavioural rhythms in the 1–8 h range and aligned physiological and biochemical processes such as gene expression (van der Veen and Gerkema, 2017; Zhu and Liu, 2023) and metabolic processing (Psomas et al., 2023).
• Circatidal rhythms expressed as ∼12.4 h rhythms in behaviours and vertical migration in marine organisms living in the tidal zone (Rock et al., 2022). These rhythms are synchronised with external cycles in ebb and flow and can be considered in both the context of “circa” and ultradian rhythms.

The expectation that, just like circadian rhythms, these rhythms are driven by a uniform and discrete oscillator mechanism is difficult to support given the range in periods between these classes. For instance, pulse generation in the HPA-axis that drives hormonal ultradian rhythms (Clarkson et al., 2017; Lightman et al., 2020) is distinctly different from central correlates of ultradian behavioural rhythms (Gerkema et al., 1990; Blum et al., 2014; Ono et al., 2015; Ono et al., 2017; Wu et al., 2018), but they generally share principles such as co-expression with circadian rhythms and variability in period and amplitude. Although the rhythm generation mechanisms are likely to differ between classes of ultradian rhythms, they may be shared amongst rhythms in the same class. Such a refined hypothesis, based on biological relationships and period similarity, can offer greater clarity and focus in the search for specific ultradian rhythm generation mechanisms—an approach that has proven successful, for example, in the study of circatidal rhythms.

Extending the definition of circadian clocks to ultradian biology also leads to the expectation that ultradian rhythm generation mechanisms are biochemically buffered (and temperature compensated), leading to a stable period despite changes in the environment or physiology. In contrast to circadian rhythms, however, ultradian rhythms are often expressed with large between-individual variation in periods, as well as non-stationary periods within an individual. For example, the period of the ultradian sleep-wake cycle in human infants varies substantially between babies and changes over early postnatal development (Löhr and Siegmund, 1999). Another example is that the frequency of reproductive hormone pulsatility changes throughout life and over the menstrual cycle (Marques et al., 2000).

These changes in period may occur in response to changes in the physiological counterparts of these ultradian rhythms and be intrinsic to the adaptive benefits ultradian rhythmicity may provide. Examples of such modulation are the experimentally induced period changes in behavioural and gene expression rhythms through altered dopamine tone or glucose concentration, respectively (Blum et al., 2014; Ting et al., 2023). This suggests that ultradian rhythm generation mechanisms may not be buffered to maintain a stable period but instead exhibit adaptive responsiveness to changes in their linked physiology.

## Why are behavioural-related circadian and ultradian rhythms linked (the phase questions)?

Almost all of the above categories of ultradian rhythms can potentially be synchronised with circadian rhythms, and there may be several functional reasons for this. Understanding these functional connections and interactions between circadian and ultradian rhythms may help provide insights into the role of ultradian rhythms in the poorly understood mechanisms through which circadian desynchrony increases the risk of developing metabolic syndrome, type-2 diabetes, and several cancer types (Kecklund and Axelsson, 2016; James et al., 2017).

The first functional reason for the synchronised co-expression of circadian and ultradian rhythms could be the synchronisation of ultradian rhythms between organisms and/or within an organism at least once per circadian cycle, as seen in behaviour. In the absence of an external counterpart for ultradian rhythms, this allows the synchronisation of behavioural ultradian rhythms to the day–night cycle (Psomas et al., 2023) and between conspecifics (Gerkema and Verhulst, 1990). Strikingly, this synchronisation of ultradian behaviour to diurnal timing is achieved through phase-dependent shifts (which can be quantified using a phase response curve (Psomas et al., 2023)), in the same way that circadian behavioural rhythms are synchronised with the day–night cycle by light (De Coursey, 1960).

The second reason for synchronised co-expression could be that it may be beneficial for ultradian rhythms to complete several full cycles every day to ensure that dependent (in)active ultradian and circadian phases can coincide. For instance, the break of fasting upon awakening is best met/anticipated by an activating prompt from both ultradian and circadian behavioural and metabolic rhythms. The observation that ultradian rhythms with periods of 12, 8, or 6 h (which are submultiples of 24 h; 24/2, 24/3, 24/4 h, etc.) coincide with circadian periods has led to some referring to them as “bimodal” or “polyphasic” circadian rhythms or rhythms resulting from intersecting circadian clocks. Whilst, from a circadian viewpoint, this may seem parsimonious, it ignores the plentiful observations that these ultradian rhythms persist in the absence of circadian clocks, as seen in SCN-lesioned or transgenic rodents, flies, and cells (Dowse et al., 1987; Gerkema et al., 1990; Schwartz and Zimmerman, 1991; Prendergast and Zucker, 2016; Goh et al., 2019; Yang et al., 2022; Zhu and Liu, 2023).

A third and more speculative theory is that ultradian rhythms may play a mechanistic role in the internal circadian dynamics and its output pathways. Can we exclude a contribution of ultradian rhythms in the timing or “gating” of protein interactions in the circadian clock? Perhaps, we have not given enough credit to the suggestion of a secondary, ultradian “bout oscillator” (Davis and Menaker, 1980) that drives discrete bouts of activity, night-time sleep staging, hormone secretion, or metabolism throughout the circadian cycle? Such a model would explain why ultradian and circadian rhythms are synchronised and why ultradian rhythms become apparent when circadian mechanisms are perturbed. Conversely, there is very little known about how the loss of ultradian rhythms impacts the expression of circadian rhythms although the loss of ultradian feeding patterns enhances the expression of circadian clocks in the livers of voles (van der Veen et al., 2006).

Whether one or multiple of these reasons for the synchronisation of circadian and ultradian rhythms could be relevant is an open question; however, it highlights that it is essential to strictly control the circadian environment when studying ultradian rhythms and recognise that an ultradian cycle observed during the day may differ significantly from one observed at night.

## Why are behavioural-related ultradian rhythms so elusive (the amplitude questions)?

Any representation of ultradian rhythmicity should thus stipulate both the physiological and circadian context in which the ultradian rhythm is expressed. This consideration is comparable to the evaluation of both homeostatic and circadian drivers under a constant routine (Duffy and Dijk, 2002), but with the critical stipulation that physiological and circadian factors can alter the period and amplitude (prominence) of ultradian rhythm expression. The circadian context, in which high-amplitude circadian rhythms mask ultradian visibility, as described above, is increasingly recognised. The exciting challenge lies in understanding the impact of physiology on the phase, period, and amplitude (i.e., prominence) of ultradian rhythms, which will also tell us which aspects of physiology are the natural intrinsic “counterpart” of ultradian rhythms.

Recently, it has become clear that behavioural ultradian rhythms are enhanced when animals experience a negative energy balance (van Oort et al., 2005; van Beest et al., 2020; van Rosmalen and Hut, 2021; Heldmaier et al., 2024). Furthermore, we have shown that ultradian feeding rhythms lead to quenching of the liver circadian clock (van der Veen et al., 2006) and are associated with ultradian metabolite rhythms in the vole (Psomas et al., 2023). This suggests that metabolism is a key linked, intrinsic counterpart of behavioural ultradian rhythms. We recently confirmed this by showing that low glucose concentration in the medium (modelling hypoglycaemia) promotes ultradian rhythmicity in mouse adipose cells in culture (van der Veen and Gerkema, 2017; Ting et al., 2023). Strikingly, this hypothesis aligns with Aschoff’s early observation that ultradian rhythms play a critical role in self-preservation of an organism by maintaining metabolic homeostasis (Aschoff and Meyer-Lohmann, 1954).

## Uncovering the functional and mechanistic link between ultradian behaviour and metabolism

**FIGURE 1:**
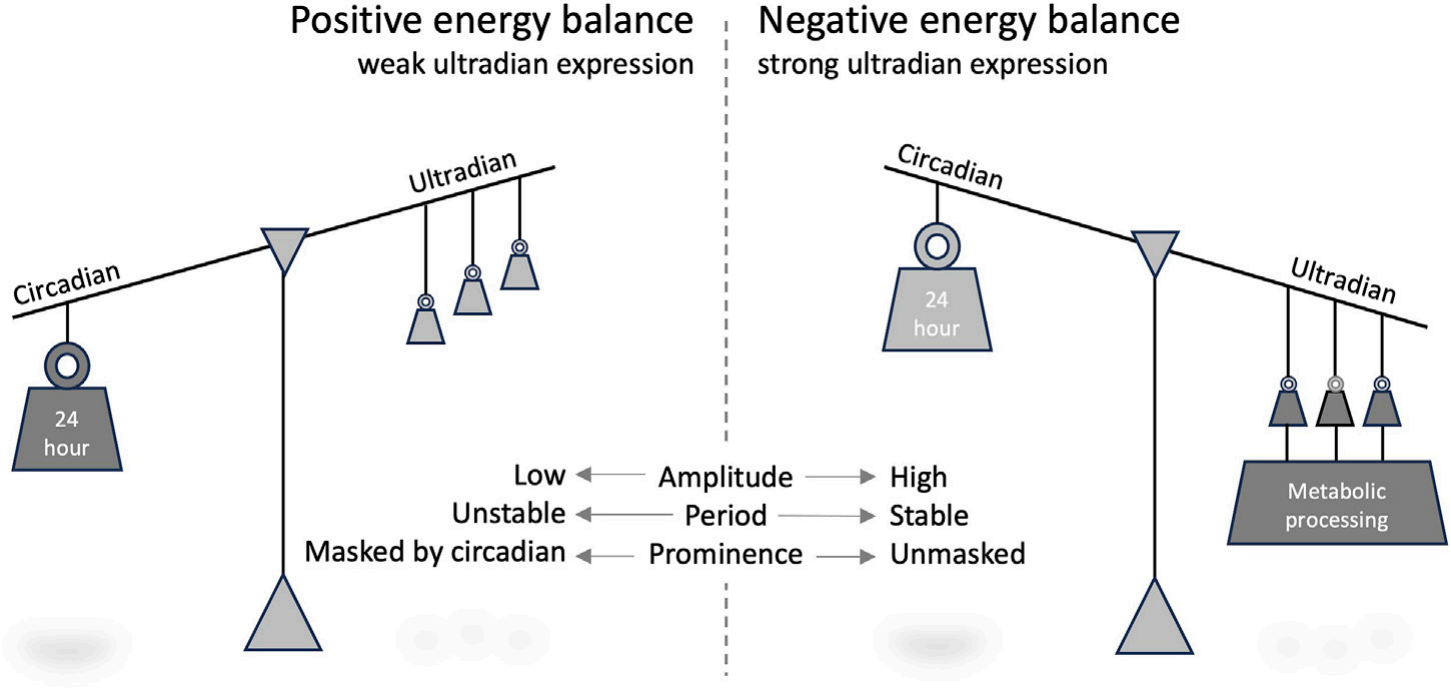
Model of the impact of energy balance on the expression of ultradian rhythms.

We posit that the amplitude and period of ultradian rhythms in behaviour and metabolic processing change in response to changed energy input (e.g., dietary energy content and food availability) or output (e.g., energy expenditure and thermogenesis). Ultradian rhythmicity amplitude is enhanced when the energy balance is negative, and/or the organism is in a fasting-like state, whereas a positive energy balance leads to ultradian rhythms being masked by circadian rhythms (Figure 1).

Moreover, given the synchronised co-expression of ultradian and circadian rhythms, enhanced ultradian rhythms lead to an overall reduced contribution of circadian rhythms and *vice versa*. Addressing this link between ultradian rhythms in metabolic processing and behaviour is now overdue, especially considering the synchronisation with circadian rhythms in the same physiological pathways, which are known to be critical to health (Kecklund and Axelsson, 2016; James et al., 2017).

Within this context, it is timely to rigorously test this hypothesis using targeted experimental manipulation of energy input or output whilst keeping circadian entrainment entirely stable. It is fundamentally important to also translate these findings to humans as it could provide crucial insights into the role of diet and energy expenditure in balancing and modulating the prominence of circadian and ultradian metabolic rhythms, an area that remains underexplored and poorly understood.
